# 

**Published:** 1888-09-22

**Authors:** 


**To Residents, Secretaries, and Matrons.**

The Editor will be glad to have the Regulations and each Report as issued by Asylums, Hospitals, Convalescent and Nursing Institutions, as well as those of other Charities, and an early copy in duplicate of all Appeals and Festival Notices, etc., addressed to *The Editor, The Lodge, Porchester Square, London, W.* Any superintendent, secretary, matron, or other chief official who regularly sends such information may be registered, on application to the Publisher, to receive free of cost a cover for binding The Hospital in half-yearly volumes.

**Notice to Advertisers and Subscribers.**

All Advertisements and Business Communications should be addressed to The Publisher, *not to the Editor*, The Hospital, 38, Old Jewry, E.C.
A Scale of Charges will be found in the Advertisement Sheets, page iii.

### A RESEARCH LABORATORY.

The question of equipping a research laboratory has for the past three years occupied a very prominent position in the discussions of the Royal College of Physicians, Edinburgh, but it was only last year that the committee appointed by the college was able to throw the plans into a feasible, and at the same time thoroughly acceptable, shape. When the matter had been taken in hand the committee entered into the scheme with great energy and thoroughness, and within a very short time suitable premises were acquired, the necessary structural alterations were at once commenced, a superintendent was appointed, and apparatus was ordered and fittings were put in hand to be ready for use as soon as the building should be prepared for their reception. The premises are well adapted for the purpose for which they were acquired. They consist of a three-storeyed house, No. 7, Lauriston Lane, near the Royal Infirmary, to which had been added a large detached room in the back court. There are also commodious out-houses and a plot of ground of considerable size at the rear of the building.

### THE CHILDREN'S CORNER.

**PRIZES.**
**FOR MOST CORRECT ANSWERS TO PUZZLES.**
**This Commenced October 1st, 1887.**
**One Pound a Month.**
**A PRIZE OF THREE GUINEAS**
**to the Competitor who shall have sent in the largest number of correct or best answers during the twelve months.**

### Fourth Week Puzzles.

No 13. DOUBLE ACROSTIC.—Two cont emporary poets.
1. "The iron Chancellor."
2. A river which gives its name to a town on its banks.
3. A favourite heroine of a favourite author.
4. A well-known writer of to-day.
5. A celebrated sculptor.

No. 14. CHARADE.

My *first* upon your table oft
At breakfast time has been;
And in your stable raised aloft
My *second* may be seen.
My *whole* contains my first in rows,
And you possess it I suppose.

No. 15. NUMERICAL ENIGMA.—I am a word of ten letters. My 1, 7, 2, 3, 4 is a sacred song; my 7, 8, 9, 6, 4 is much dreaded by my 7, 5, 2, 4, 5, 10; my 1, 2, 6, 2, 7, 9, 3 is much used by ladies; my 6, 9, 7, 5 is a flower; my 1, 9, 8, 2, 8, 9, 5 is a vegetable; my 6, 9, 4, 5 is an ancient city my 7, 9. 3, 9. 4, 9, 10 a wise king.

No. 16. DECAPITATION.—I am a weight; behead me I am an animal behead me again and I am part of a verb.

### Results of Second Week Puzzles.

No. 5. DOUBLE ACROSTIC.

C omi C
U proa R
R os E
D ahli A
S tea M

No. 6. ANAGRAMS.
1. Thomas Carlyle.
2. Victoria Regina.
3. Sir Edwin Landseer.
4. Joseph Hume.

No. 7. BURIED BIRDS.—Condor. Linnet. Ostrich.

No. 8. ENIGMA.—Corn.

| Marks. | Sept. 8th. | Total First and Second Weeks. |
|---|---|---|
| A. C. K. | 3 | 7 |
| Gem | 2 | 6 |
| G. M. M. | 1 | 4 |
| Scrooge | — | 3 |
| Thanet | 3 | 7 |
| Tynesider | — | 3 |
| Judy | — | 3 |
| Primrose | 4 | 7 |
| J. W. M. | 1 | 4 |
| Agamemnon | 4 | 8 |
| G. E. R. | 3 | 6 |
| Happy Eliza | — | 3 |
| Penelope | 1 | 4 |
| Ikye Mo | — | 1 |
| Boadicea | 2 | 6 |

